# A Multicenter, Retrospective Analysis of Long‐Term Survival in 255 Dogs With Pheochromocytoma Treated With Alpha‐Adrenoreceptor Antagonists or Surgery (2010–2021)

**DOI:** 10.1111/jvim.70220

**Published:** 2025-09-01

**Authors:** Matthew M. E. Steele, Marit F. van den Berg, Sara Galac, Ana M. Dordio, Anna Threlfall, Cláudia Gomes, Amanda Paul, Mirja Nolff, Nadja Sieber‐Ruckstuhl, Ghita Benchekroun, Fergus Allerton, Beatriz Mendoza, Susanna Spence, Paula Valiente, Alisdair Boag, Federico Fracassi, Alejandra Carranza Valencia, Jorge Pena‐Ramos, Ben Lloyd‐Bradley, Mayank Seth, Jack Fawsitt, Fang Yu Foo, Romy M. Heilmann, Paolo Silverstrini, Aurélie Lyssens, Emilie Vangrinsven, Clara Casas‐Acuna, Alenka Hrovat, Carolina Arenas, Mario Cervone, Carmel T. Mooney, Rodolfo O. Leal, Karolina Maria Piekos, Gerard Olivares, Guillame Ruiz, Sarah J. Caulfield, Sophie Keyte, Anaïs Lamoureux, Christopher J. Scudder, Ruth Gostelow

**Affiliations:** ^1^ ChesterGates Veterinary Specialists Chester UK; ^2^ Clinical Science and Services Royal Veterinary College London UK; ^3^ Department of Clinical Sciences, Faculty of Veterinary Medicine Utrecht University Utrecht the Netherlands; ^4^ Davies Veterinary Specialists Hitchin UK; ^5^ Anderson Moores Veterinary Specialists Winchester UK; ^6^ The Queens Veterinary School Hospital University of Cambridge Cambridge UK; ^7^ Hamilton Specialist Referrals Wycombe UK; ^8^ Clinic for Small Animal Surgery, Vetsuisse Faculty University of Zurich Zurich Switzerland; ^9^ Clinic for Small Animal Internal Medicine, Vetsuisse Faculty University of Zurich Zurich Switzerland; ^10^ Ecole Nationale Vétérinaire d'Alfort, CHUVA, Unité de Médecine Interne Maisons‐Alfort France; ^11^ Willows Veterinary Specialists Shirley UK; ^12^ North Downs Specialist Referrals Bletchingley UK; ^13^ London Vet Specialists London UK; ^14^ Veterinary Specialty Hospital of Hong Kong Kowloon People's Republic of China; ^15^ Royal (Dick) School of Veterinary Studies University of Edinburgh Edinburgh UK; ^16^ Department of Veterinary Medical Sciences University of Bologna Bologna Italy; ^17^ Department of Clinical Veterinary Science University of Bern Bern Switzerland; ^18^ Small Animal Referral Hospital, Langford Vets University of Bristol Bristol UK; ^19^ Dick White Referrals Cambridge UK; ^20^ Stansted Veterinary Specialists Bishop's Stortford UK; ^21^ Faculty of Veterinary Medicine Universität Leipzig Leipzig Germany; ^22^ Small Animal Teaching Hospital University of Liverpool Liverpool UK; ^23^ Ryan Veterinary Hospital University of Pennsylvania Philadelphia Pennsylvania USA; ^24^ Faculty of Veterinary Medicine University of Liège Liège Belgium; ^25^ Pride Veterinary Referrals Derby UK; ^26^ AniCura Valencia Sur Valencia Spain; ^27^ Sevetys Crozatier Paris France; ^28^ University College Dublin Veterinary Hospital Dublin Republic of Ireland; ^29^ Centre for Interdisciplinary Research in Animal Health/Associate Laboratory for Animal and Veterinary Sciences (AL4AnimalS), Faculty of Veterinary Medicine University of Lisbon Lisbon Portugal; ^30^ Eastcott Veterinary Referrals Swindon UK; ^31^ Bristol Vet Specialists Bristol UK; ^32^ Lumbray Park Veterinary Specialists Alton UK; ^33^ Centre Hospitalier Vétérinaire Anicura Nordvet La Madeleine France

**Keywords:** adrenal, adrenalectomy, endocrine, metanephrine, phenoxybenzamine

## Abstract

**Background:**

The survival of dogs with pheochromocytoma (PCC) treated with adrenoreceptor antagonists has not been described or compared to surgically managed cases.

**Hypothesis/Objectives:**

The objective of this study is to evaluate the survival of medically and surgically managed dogs with PCC and investigate factors associated with survival.

**Animals:**

Two hundred fifty‐five dogs with PCC, treated with alpha‐adrenoreceptor antagonists (AA) without adrenalectomy (Group 1, *n* = 75), adrenalectomy +/– AA (Group 2, *n* = 128), or neither treatment (Group 3, *n* = 52).

**Methods:**

Retrospective, multicenter review of medical records. Median overall survival time (OST) for Groups 1 and 2 combined was calculated using Kaplan–Meier estimates, and then compared between Group 1 and Group 2 using Log‐Rank testing. Cox proportional hazard analysis identified factors associated with survival in Groups 1 and 2 individually and combined.

**Results:**

Median OST for all cases was 854 (95% CI: 572–1136) days. Median OST was lower in Group 1 (247 days, 95% CI: 76–418 days) than in Group 2 (927 days, 95% CI: 587–1267 days; *p* < 0.001). In Group 2, 88/92 dogs (97.8%) that received presurgical AA treatment survived to discharge compared to 23/27 (85.2%) that did not receive AA pretreatment (*p* = 0.03). Lack of clinical signs at presentation was associated with increased survival in both groups combined (HR 0.5; 95% CI 0.3–0.9; *p* = 0.02) and in Group 2 alone (HR 0.3; 95% CI 0.1–0.7; *p* = 0.01).

**Conclusions and Clinical Importance:**

Dogs with PCC treated with adrenalectomy have longer survival compared to those managed with AA without adrenalectomy.

**Abbreviations**
[131]‐IMBG: Iodine‐131 metaiodobenzylguanidine
AA: alpha‐adrenoreceptor antagonist
COX: Cox proportional hazard analysis
H&E: hematoxylin and eosin
IHC: immunohistochemistry
KM: Kaplan–Meier
LR: logistic regression
OR: odds ratio
OST: overall survival time
PBZ: phenoxybenzamine
PCC: pheochromocytoma
pNMN: plasma‐free normetanephrine
PRZ: prazosin
rTKI: receptor‐tyrosine kinase inhibitor
SRT: stereotactic radiation therapy
uNMN:Cr: urinary normetanephrine to creatinine ratio

## 1 Introduction

Pheochromocytoma (PCC) is a tumor of the adrenal medullary chromaffin cells with a reported prevalence of 0.01% to 0.1% in dogs referred for evaluation [1, 2]. PCCs can result in a wide range of clinical signs that make diagnosis and treatment challenging [1, 2, 3, 4, 5].

Historical case series of dogs with PCCs have predominantly used postmortem diagnoses [1, 2]. The availability of advanced imaging and measurement of urine or plasma catecholamine metabolites has likely made antemortem diagnosis more common [6, 7, 8, 9, 10, 11, 12]. Urinary normetanephrine:creatinine ratio (uNMN:Cr) distinguishes healthy dogs and dogs with hypercortisolism from those with PCC when a cutoff of > 4 times the upper limit of the reference interval is used. Sensitivity and specificity data for this analyte have not been reported [8, 10]. In contrast, plasma‐free normetanephrine (pNMN) has a sensitivity of 62.5%–100% and specificity of 94%–100% for the diagnosis of PCC in dogs [6, 7]. A group of dogs with PCC that has been diagnosed based on metanephrine testing antemortem, without adrenal histopathology to confirm the diagnosis, has not been reported in the veterinary literature.

The treatment of choice for nonmetastatic functional PCC in dogs is generally considered to be adrenalectomy [3, 4, 13]. Other described treatments include stereotactic radiation therapy (SRT) and receptor‐tyrosine kinase inhibitors (rTKI) and a single case of treatment using iodine‐131 metaiodobenzylguanidine ([131]‐IMBG) [14, 15, 16]. Alpha‐adrenoreceptor antagonists (AA) have been used in the preoperative stabilization of dogs with PCC [3, 4, 5]. The evidence for AA use in this context is limited to a single study and has not been replicated in subsequent studies [17, 18, 19, 20]. It has been proposed that treatment of dogs with PCC that are not managed surgically can include longer‐term use of AAs [3, 5]. Anecdotally, it appears that this option is utilized in clinical practice relatively frequently if more definitive treatment options are not considered appropriate or are declined by owners. There are currently no studies reporting this treatment approach in dogs with PCC. Furthermore, risk factors for long‐term survival of dogs with PCC are largely based on surgical studies, and no literature exists reporting the outcomes or risk factors for long‐term survival in dogs with PCC that are not treated by adrenalectomy. Although a recent study has suggested that the survival of dogs with invasive adrenal tumors not treated surgically is poor, anecdotal experience suggests that some dogs treated with AA alone have good outcomes [21].

The primary aim of this study was to describe dogs with PCC treated by adrenalectomy or with AA management alone and compare the survival times of these groups. A secondary aim was to evaluate risk factors associated with survival in these populations combined and individually. It was hypothesized that cases managed solely with AA have a comparable survival time to surgically managed cases, with or without AA pretreatment.

## 2 Methods

Ethical approval for the study was granted by the Social Science Research Ethical Review Board (SSREB), Royal Veterinary College: URN SR2020‐024. Data were collected from dogs that had presented to referral centers in Europe between January 01, 2010 and December 31, 2021. Inclusion criteria were: dogs with a histopathologic diagnosis of PCC (definitive on hematoxylin and eosin [H&E] staining, or suspected on H&E and subsequently confirmed with immunohistochemistry [IHC]), or dogs with a clinical diagnosis based on supportive clinical and clinicopathologic findings, adrenal enlargement identified using abdominal imaging and either pNMN or uNMN:Cr compatible with a diagnosis of PCC. A cutoff of pNMN concentration of ≥ 5.52 nmol/L was used due to its specificity of 97.6% and similarity in laboratory methods used during the time period of this study [6]. For uNMN:Cr, a value of ≥ 4× the upper reference interval was used [8, 10]. If a laboratory reference interval was not available, or if a human laboratory was used for uNMN:Cr measurement, then an arbitrary cutoff value of uNMN:Cr of ≥ 400 (nmol/mmol) was used. These values are based on those that distinguish dogs with PCC from healthy dogs, non‐adrenal illness, and adrenocortical tumors from available literature [8, 9, 10, 11].

Cases were excluded if inclusion criteria were not met. Cases were also excluded if adrenal histopathology was not available and they had received medications likely to interfere with metanephrine testing, based on guidelines from human literature (including corticosteroids, AA, beta‐blockers, calcium channel blockers, metoclopramide, serotonin reuptake inhibitors, sulfasalazine, sympathomimetic medications, and tricyclic anti‐depressants). Cases were also excluded if there was a suspicion of dogs with Cushing's syndrome in the clinical history or, when performed, an adrenocorticotropin (ACTH) stimulation test or low‐dose dexamethasone suppression test (LDDST) that could be compatible with hypercortisolism [22]. To reduce the likelihood of inadvertently including cases with Cushing's syndrome, either laboratory‐specific reference intervals, where available, or current veterinary clinical guidelines for the testing of endocrine disease were used to exclude these cases [23].

Contributing veterinarians from each center uploaded data from suitable cases using an online secure database (Castor Electronic Data Capture [CastorEDC], available at https://www.castoredc.com). Data collected included patient signalment, clinical history, physical examination findings, and clinicopathologic data. Results from cardiac evaluation, thoracic radiography and abdominal ultrasonography, computed tomography and magnetic resonance imaging, adrenal cytology, endocrine test results including metanephrine testing, and histopathology with or without IHC were also collected where available. An imaging category for possible thoracic or abdominal metastatic disease was included. Blood pressure was recorded as the maximum and minimum systolic readings documented on presentation, and categorization of measurements according to risk of target organ damage was based on the minimum blood pressure measurements [23]. Information on treatment (including AA drug, treatment frequency, minimum and maximum doses), surgical factors (including preoperative AA drugs, doses and frequencies, location and extent of vascular invasion, renal capsular invasion, requirements for venotomy, and whether a nephrectomy was performed), postoperative complications, and outcome were collected where possible. Outcomes recorded included the date of last contact with the owner, causes of death, death during anesthesia, and date of discharge after surgery.

Data were entered into the online database, were anonymized, and then individually reviewed by the primary authors (MMES, CJS, RG) to ensure that cases met the inclusion criteria and that there were no discrepancies in data entry prior to the generation of descriptive data and statistical analysis. Included cases were divided based on whether they were treated with AA without adrenalectomy (Group 1), by adrenalectomy with or without AA (Group 2), and those that were not treated with either of these modalities (Group 3).

### 2.1 Statistical Analysis

Statistical analyses were performed using SPSS Statistics v28.0 for Mac (IBM Corporation, New York, USA). Normality of distribution of continuous data was assessed by visual inspection of histograms and Shapiro–Wilk tests. Data are presented as median (range) or mean (±SD), depending on normality. The frequency of cases surviving to discharge in the surgical group (Group 2) receiving AA pretreatment was compared with those that did not receive AA pretreatment using a Fisher's exact test. Binary logistic regression (LR) was used to evaluate for any factors that might have biased toward selection of cases for AA management alone versus management with adrenalectomy. Factors included in LR included age at presentation, the total and individual numbers of comorbidities, maximum tumor size identified on any imaging modality, whether the tumor was left or right sided, presence of vascular invasion, presence of suspected/confirmed thoracic or abdominal metastasis, and blood pressure at presentation. Contingency table analysis using Monte–Carlo simulation was performed to evaluate whether certain centers had a bias in selection of cases between AA and surgical treatment. Survival analyses are presented with Kaplan–Meier (KM) curves. The survival times of Group 1 and Group 2 were compared using log‐rank testing, and risk factors for survival were assessed using Cox proportional hazard regression. Death due to any cause (all‐cause mortality) was treated as an event. Survival times are presented as overall survival time (OST). Cases alive at the time of last contact were censored at that timepoint. Univariable Cox‐regression (COX) was performed on variables considered to be biologically relevant [24]. Variables with missing data (> 20%) were either excluded from analysis or a separate category called “not reported” was created to allow inclusion of variables deemed to be of higher clinical importance (such as tumor extension beyond the diaphragm). Clinical categorical variables (e.g., presenting signs) were included if they affected > 10% of the group. Where considered most biologically relevant, continuous data without infinite scales were converted to categorical variables (e.g., temperature was converted to normo‐, hypo‐, and hyper‐thermia). Factors of interest in univariable modeling were identified via exclusion of variables not reaching *p* < 0.1. Furthermore, variables giving duplicate information were excluded dependent on the degree of missing data or based on the variable that was of most clinical relevance. After variable selection, multivariable COX modeling was performed on the combined data from Group 1 and Group 2 cases initially. Given the presence of factors within each group that would be unique to those individual populations, further individual models for Group 1 and Group 2 were also built. All multivariable COX models were generated using systematic manual backward‐stepwise inclusion. Variables with the lowest significance or greatest amounts of missing data, resulting in instability of the models, were excluded at each step until a significance of *p* < 0.05 for all remaining variables was reached.

## 3 Results

### 3.1 Study Groups

A total of 364 cases were entered into the online database from 26 referral centers. Following case review, 255 (70.1%) cases were included (Table S1). Of these cases, 58.4% (*n* = 149/255) had a histopathological diagnosis of PCC, and the remaining 41.5% (*n* = 106/255) had clinical diagnoses.

**FIGURE 1:**
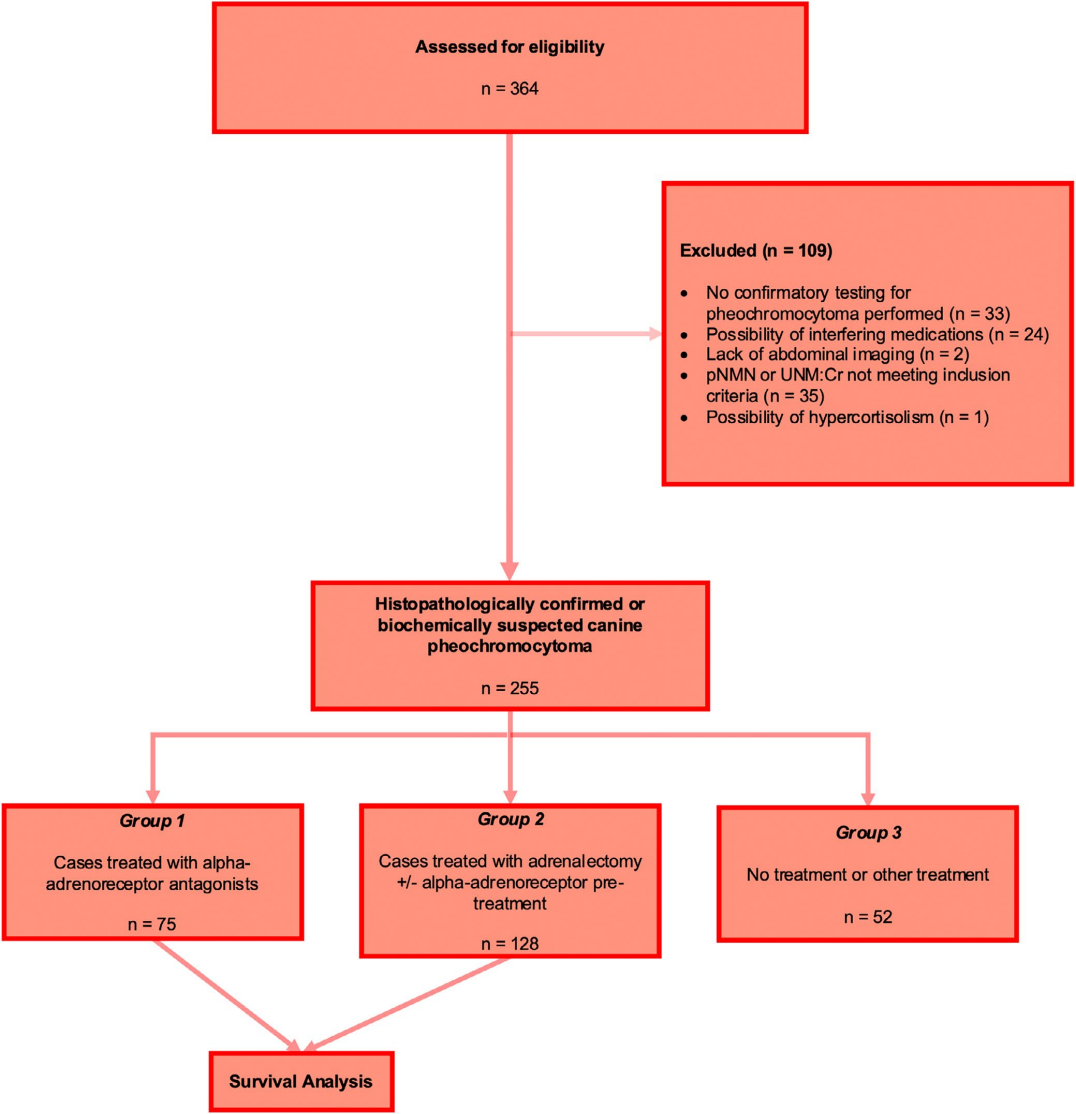
STROBE diagram illustrating number of cases assessed for eligibility, and details of reasons for exclusion and division of groups used in the descriptive portion of the study as well as in survival analysis.

Group 1 consisted of 29.4% (*n* = 75/255) cases treated with an AA without adrenalectomy. Group 2 consisted of 50.2% (*n* = 128/255) cases that underwent adrenalectomy with or without AA pretreatment, and Group 3 consisted of 20.4% (*n* = 52/255) cases that either did not receive treatment or were treated with medications other than AA. This final group was not included in the survival analysis. A breakdown of included and excluded cases, along with numbers that entered the descriptive and survival analysis portions of the study, is presented in Figure 1. Further information and descriptive statistics detailing demographic data, historical complaints, and results of initial investigations for these groups are presented in Tables S2–S5 and Figures S1–S3. Treatment Groups.

#### 3.1.1 Group 1

Within Group 1, 87% (*n* = 65/75) cases were treated with phenoxybenzamine (PBZ) and the remaining 13% (*n* = 10/75) cases with prazosin (PRZ). No cases received beta‐blockers. Information regarding initial doses of PBZ and dosing frequency was available in 63 of 65 cases, with a median starting dose of 0.3 (range 0.2–1.9) mg/kg. Four of 63 cases (6%) were treated once daily, 58/63 (92%) twice daily, and one case was treated three times daily. Progressive dose increases were made in 10 PBZ‐treated cases.

All cases treated with PRZ had starting doses and dose frequency information recorded. The median starting dose was 0.1 (range 0.1–1.0) mg/kg. Dosing frequency was once daily in one case, twice daily in six cases, and three times daily in three cases. Three dogs were noted to have had dose increases made up to a median of 0.2 (range 0.1–2.8) mg/kg.

#### 3.1.2 Group 2

Of the 128 cases that were treated via adrenalectomy, 71.9% (*n* = 92/128) cases were pretreated with an AA. Further information regarding surgical pretreatment variables is presented in Table S6.

Vascular invasion was documented in 41.4% (*n* = 53/128) cases, and venotomy was performed in 83% (*n* = 39/53) of these. Renal capsular invasion was reported at surgery in 10.2% (*n* = 13/128) dogs. Nine (7.0%) of 128 cases had a nephrectomy performed. Tumor extension beyond the diaphragm was reported in 3.1% (*n* = 4/128) of dogs.

Postoperative complications were reported in 31.2% (*n* = 40/128) dogs. The frequency of individual complications is reported in Table S7. All cases survived general anesthesia. Death before discharge was recorded in 6.3% (*n* = 8/128) of dogs in Group 2. Of the cases in which the use or nonuse of surgical pretreatment with AA and outcome after surgery was known (*n* = 117/128), the frequency of survivors to discharge was significantly higher (98%, *n* = 88/90) in cases that were pretreated compared to those that were not pretreated (85%, *n* = 23/27; *p* = 0.03).

#### 3.1.3 Group 3

Descriptions of outcomes in Group 3 cases are presented in Data S1. Given inherent bias in the follow‐up of this group (i.e., high proportion lost to follow‐up or euthanized within a short time frame), survival analysis for this group was not considered appropriate. Subsequent sections of this manuscript, therefore, refer to cases in Group 1 and Group 2 only.

### 3.2 Evaluation for Treatment Selection Bias

Univariable binary LR results for clinical factors that might have resulted in treatment selection bias are presented in Table 1. Age at presentation and the adrenal gland affected were carried forward to multivariable binary LR. Age at presentation remained a significant factor, with older animals having reduced odds of selection for surgical treatment (OR 0.785, 95% CI: 0.670–0.919, *p* < 0.01; *X* ^2^ = 20.617, *p* < 0.001, H&L 0.891). The median age at presentation was 11.0 (range, 5.0–15.0) years in Group 1 versus 10.0 (range, 4.0–14.0) years in Group 2.

**TABLE 1:** Univariable binary logistic regression to evaluate factors that may have biased treatment selection.

	Number of cases (total 203)	Odds ratio	95% confidence interval	*p*
Age at presentation	199	0.799	0.690–0.925	< 0.01
Adrenal gland affected				
Right (ref)	84			0.03
Left	106	0.699	0.380–1.288	0.38
Bilateral	8	0.061	0.007–0.518	0.01
CVC invasion				
No	117			
Yes	86	1.069	0.600–1.905	0.82
Possible abdominal metastasis				
No	181	2.144	0.757–6.073	0.15
Yes	22			
Maximum tumor diameter (mm)	182	1.001	0.997–1.006	0.61
Comorbidities				
Absent	189			
Present	14	0.562	0.189–1.670	0.30
Presence of hypertension				
No	64			
Yes	43	0.631	0.283–1.411	0.26

Contingency table analysis identified two centers with significant differences in case numbers between AA and surgical treatment groups (Fisher's exact 40.948, *p* = 0.01). One center had an increased frequency of cases having surgical management, and one center was found to have an increased frequency of cases receiving AA treatment. On follow‐up with these centers, the reason for biased selection toward surgical management was due to clinician preference. In the center with a bias toward medical management, this was suspected to be due to the financial implications of surgery and regional surgeon availability. Neither of these findings was thought to preclude their inclusion in subsequent analyses.

### 3.3 Causes of Death

Information on causes of death was limited and available in 53% (*n* = 40) cases within Group 1 and 26% (*n* = 28) cases within Group 2. Details on recorded causes of death in these cases are available in Table S8.

### 3.4 Overall Survival

In Group 1, the median time from presentation to initiation of AA treatment was 14 (range 0–218) days, and the median time between presentation and adrenalectomy in Group 2 was 32 (0–694) days. Six cases (4.7%) in Group 2 (2 cases with missing data) had surgery on the day of presentation. In one case, the exact date of presentation to the referral center was not recorded.

**FIGURE 2:**
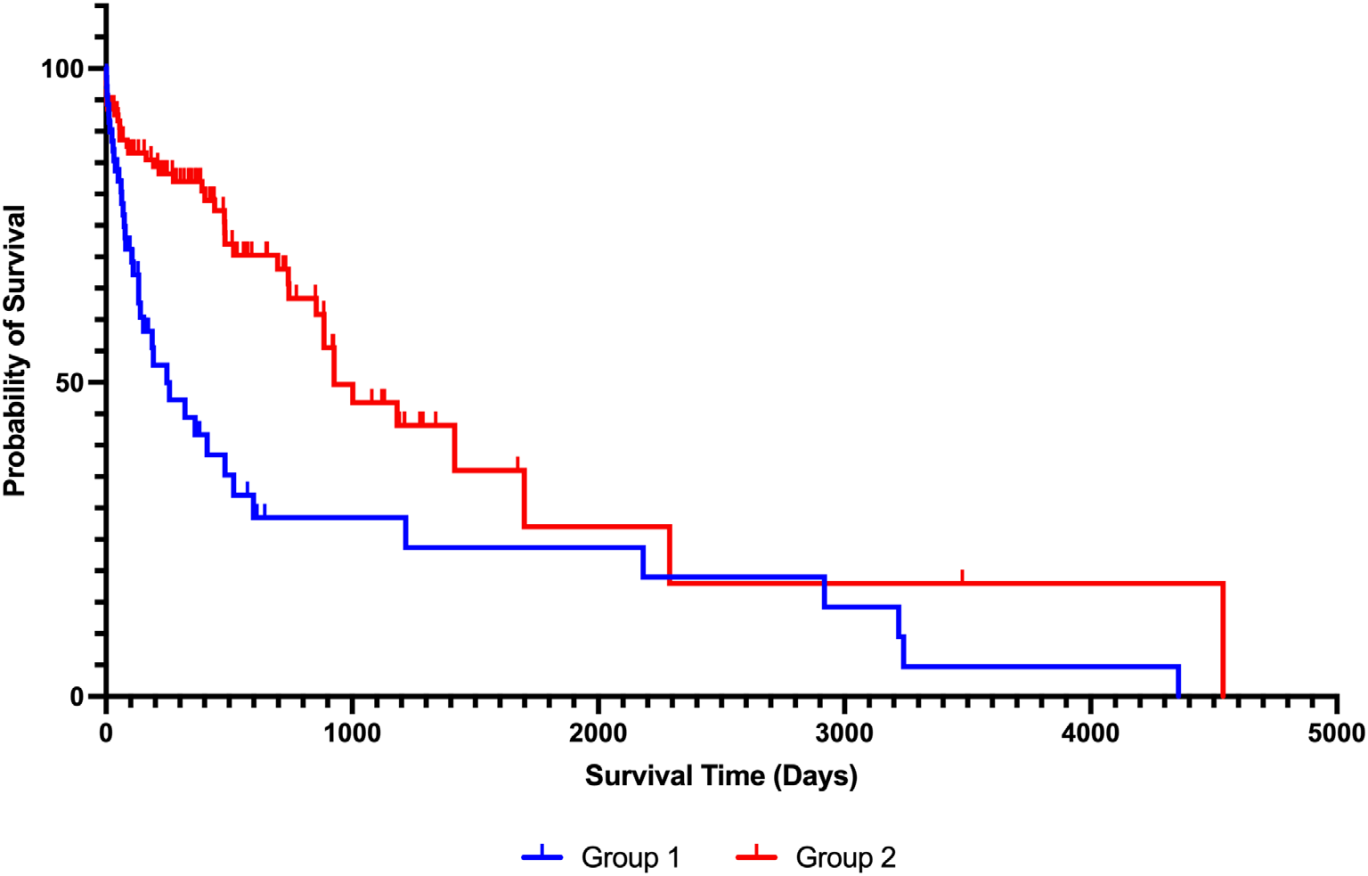
Kaplan–Meier estimate of overall survival for Group 1 (adrenoreceptor‐antagonist treated) and Group 2 (surgically treated) cases of dogs with PCC. Vertical ticks represent censoring, and vertical continuous lines represent events.

Kaplan–Meier survival curves for Group 1 versus Group 2 cases are presented in Figure 2. The median OST was 854 (95% CI: 572–1136) days for Group 1 and 2 combined. Median survival of Group 2 (927, 95% CI: 587–1267 days) was significantly longer than Group 1 (247, 95% CI: 76–418 days; *p* < 0.001).

### 3.5 Risk Factors for Survival: Groups 1 and 2 Combined

Univariable comparisons of risk factors for survival of Group 1 and Group 2 combined are presented in Table 2. Factors selected for inclusion in the multivariable model included age at presentation, history of exercise intolerance, history of weight loss, panting at presentation, heart rate at presentation, the absence of clinical examination abnormalities at presentation, serum alanine aminotransferase (ALT) activity, serum total protein concentration, evidence of abdominal metastatic disease documented via diagnostic imaging, and treatment group. The final multivariable model is presented in Table 3.

**TABLE 2:** Univariable Cox proportional hazard analysis of risk of death (all‐cause) for Group 1 and Group 2 combined.

	Number of cases	Hazard ratio	95% confidence interval	*p*
Age of presentation (years)	192	1.20	1.06–1.35	< 0.01^a^
Sex	196			
FN (ref)	71			0.16
MN	78	1.28	0.76–2.16	0.35
FE	10	2.40	0.98–5.88	0.06
ME	37	0.85	0.44–1.64	0.63
Duration of clinical signs	188			
Incidental finding (ref)	46			0.96
Day of presentation	8	0.95	0.22–4.17	0.95
< 1 week	50	1.14	0.59–2.23	0.70
1–2 weeks	15	0.80	0.31–1.99	0.63
2–4 weeks	20	1.16	0.49–2.76	0.73
> 4 weeks	49	0.91	0.47–1.78	0.79
Coughing	196			
No (ref)	185			
Yes	11	1.16	0.55–2.42	0.70
Diarrhea	196			
No (ref)	172			
Yes	24	0.80	0.40–1.60	0.52
Exercise intolerance	196			
No (ref)	174			
Yes	22	1.80	0.96–3.38	0.07^a^
Polydipsia/polyuria	196			
No (ref)	137			
Yes	59	0.93	0.57–1.53	0.78
Weight loss	196			
No (ref)	171			
Yes	25	2.09	1.13–3.84	0.02^a^
Vomiting (< 3 weeks)	196			
No (ref)	156			
Yes	40	1.33	0.79–2.26	0.29
Rectal temperature (°C)	135			
37.7–39.2 (ref)	111			0.17
< 37.7	16	1.90	0.96–3.75	0.06
> 39.2	8	0.95	0.29–3.14	0.93
Heart rate (bpm)	167			
60–120 (ref)	122			
> 120	45	0.48	0.25–0.91	0.024^a^
Respiratory rate (breaths/min)	97	0.99	0.97–1.02	0.51
Panting (presentation)	196			
No (ref)	137			
Yes	59	0.65	0.39–1.07	0.09^a^
Bodyweight (kg)	190	1.01	0.99–1.03	0.41
NIBP (mmHg)	104			
< 80	4			0.74
80–119	13	0.69	0.17–2.89	0.61
120–139	20	0.41	0.10–1.68	0.13
140–159	26	0.57	0.16–2.08	0.40
160–179	17	0.40	0.10–1.64	0.20
≥ 180	24	0.67	0.18–2.47	0.55
Abdominal distension	196			
No (ref)	173			
Yes	23	1.83	0.83–4.00	0.13
Abdominal pain	196			
No (ref)	160			
Yes	36	1.89	1.10–3.05	0.02
Heart murmur	196			
No (ref)	153			
Yes	43	0.77	0.44–1.35	0.35
No physical examination abnormalities	196			
No (ref)	133			
Yes	63	0.05	0.31–0.86	0.01^a^
Anemia	169			
No (ref)	141			
Yes	28	1.11	0.61–2.03	0.73
WBC (×10^9^/L)	164	1.00	0.98–1.04	0.79
HCT (%)	175	0.99	0.96–1.02	0.44
PLT (×10^9^/L)	159	1	1.00–1.00	0.99
ALP (U/L)	164	1	1–1	0.67
ALT (U/L)	168	1.00	1–1.00	0.03^a^
Bilirubin (mg/dL)	133	0.39	0.11–1.46	0.16
Chloride (mmol/L)	144	0.97	0.92–1.02	0.25
Creatinine (mg/dL)	180	1.42	1.12–1.79	< 0.01
Glucose (mg/dL)	138	1.00	0.99–1.01	0.93
Potassium (mmol/L)	168	0.79	0.52–1.19	0.26
Sodium (mmol/L)	167	0.98	0.93–1.03	0.39
Total calcium (mg/dL)	151	1.13	0.90–1.42	0.30
Total protein (g/L)	170	1.02	1.00–1.05	0.08^a^
pNMN (nmol/L)	73	1.00	1.00–1.00	0.14
uNMN:Cr (nmol/mmol)	68	1	1–1	0.64
CVC invasion	196			
No (ref)	114			
Yes	82	1.13	0.72–1.78	0.59
Possible abdominal metastasis	196			
No (ref)	177			
Yes	19	2.01	0.91–4.46	0.09^a^
Maximum tumor diameter (mm)	175	1.00	1.00–1.00	0.60
Thromboembolic disease	196			
No (ref)	160			
Yes	36	0.76	0.40–1.44	0.40
Treatment group	196			
Group 2 (ref)	125			
Group 1	71	2.36	1.50–3.72	< 0.001^a^
Treatment with chemotherapy agents	193			
No (ref)	173			
Yes	20	1.26	0.63–2.55	0.51

Abbreviations: ALP, alkaline phosphatase; ALT, alanine aminotransferase; bpm, beats per minute; FE, female entire; FN, female neutered; Group 1, adrenoreceptor‐antagonist treated; Group 2, surgically treated; HCT, hematocrit; ME, male entire; MN, male neutered; NIBP, noninvasive blood pressure; PLT, platelet count; pNMN, plasma normetanephrine; uNMN:Cr, urinary normetanephrine to creatinine ratio; WBC, white blood cell count.

^a^ Variables selected for multivariable analysis.

**TABLE 3:** Multivariable Cox proportional hazard analysis of risk of death (all‐cause) for Group 1 and Group 2 combined.

	Hazard ratio	95% confidence interval	*p*
No physical examination abnormalities			
No (ref)			
Yes	0.48	0.26–0.87	0.02
Panting at presentation			
No (ref)			
Yes	0.55	0.32–0.96	0.03
Weight loss			
No (ref)			
Yes	3.26	1.58–6.73	< 0.01
Total protein (g/L)	1.03	1.01–1.06	0.02
Treatment group			
Group 1 (ref)			
Group 2	0.39	0.23–0.67	< 0.001

Abbreviations: Group 1, adrenoreceptor‐antagonist treated; Group 2, surgically treated.

The groups were subsequently analyzed independently, given that some variables might have been unique to each group. Univariable results are presented in Table 4 for Group 1. Factors selected for multivariable modeling were duration of clinical signs, historical polyuria/polydipsia (PU/PD), and acute vomiting; the presence of a heart murmur on clinical examination; white blood cell count; serum ALT activity; serum bilirubin; creatinine; potassium; and total protein concentration. The multivariable model for this group is presented in Table 5.

**TABLE 4:** Univariable Cox proportional hazard analysis of risk of death (all‐cause) for Group 1.

	Number of cases	Hazard ratio	95% confidence interval	*p*
Age of presentation	68	1.03	0.87–1.22	0.76
Sex	71			
FN (ref)	23			0.16
MN	27	1.54	0.70–3.38	0.28
FE	4	3.69	1.01–13.58	0.05
ME	17	0.87	0.36–2.11	0.75
Duration of clinical signs	66			
Incidental finding (ref)	14			0.05^a^
Day of presentation	2	2.82	0.33–23.92	0.34
< 1 week	21	0.96	0.40–2.73	0.94
1–2 weeks	3	8.07	1.86–35.04	0.05
2–4 weeks	7	1.79	0.50–6.41	0.37
> 4 weeks	19	0.98	0.36–2.66	0.96
Collapse episodes	71			
No (ref)	58			
Yes	13	0.62	0.28–1.38	0.24
Coughing	71			
No (ref)	63			
Yes	8	0.75	0.31–1.81	0.52
Diarrhea	71			
No (ref)	59			
Yes	12	1.11	0.48–2.56	0.82
Polydipsia/polyuria	71			
No (ref)	47			
Yes	24	2.08	1.08–4.00	0.03^a^
Vomiting (acute)	71			
No (ref)	57			
Yes	14	3.64	1.69–7.85	< 0.01^a^
Rectal temperature (°C)	50			
37.7–39.2 (ref)	40			0.49
< 37.7	6	0.78	0.44–1.39	0.40
> 39.2	4	0.71	0.39–1.31	0.27
Heart rate (bpm)	65			
60–120 (ref)	44			
> 120	21	0.65	0.30–1.44	0.293
Respiratory rate	41	1.00	0.96–1.02	0.50
Panting (presentation)	71			
No (ref)	47			
Yes	24	0.68	0.34–1.35	0.27
Bodyweight (kg)	70	1.01	0.99–1.04	0.26
NIBP (mmHg)	36			
< 80	1			0.11
80–119	4	0.04	0.00–0.75	0.03
120–139	6	0.02	0.00–0.41	0.01
140–159	8	0.03	0.00–0.62	0.02
160–179	9	0.01	0.00–0.27	0.01
≥ 180	8	0.04	0.00–0.76	0.03
Abdominal distension	71			
No (ref)	62			
Yes	9	1.32	0.54–3.20	0.54
Abdominal pain	71			
No (ref)	57			
Yes	14	1.35	0.70–2.82	0.42
Heart murmur	71			
No (ref)	55			
Yes	16	0.46	0.21–1.01	0.054^a^
No physical examination abnormalities	71			
No (ref)	54			
Yes	17	0.7	0.30–1.42	0.28
Anemia	58			
No (ref)	46			
Yes	12	1.11	0.72–1.69	0.643
WBC (×10^9^/L)	57	1.08	1.04–1.13	< 0.001^a^
HCT (%)	61	1.00	0.95–1.03	0.62
PLT (×10^9^/L)	53	1.00	1.00–1.00	0.55
ALP (U/L)	56	1	1–1.00	0.67
ALT (U/L)	58	1.00	1–1.00	0.06^a^
Bilirubin (mg/dL)	45	28.60	0.59–1379.19	0.09^a^
Chloride (mmol/L)	51	0.93	0.85–1.03	0.15
Creatinine (mg/dL)	68	1.45	1.14–1.84	< 0.01^a^
Glucose (mg/dL)	51	1.01	0.99–1.03	0.27
Potassium (mmol/L)	64	0.53	0.28–1.02	0.06^a^
Sodium (mmol/L)	63	0.95	0.87–1.02	0.16
Total calcium (mg/dL)	53	1.08	0.78–1.49	0.66
Total protein (g/L)	58	1.04	1.00–1.08	0.06^a^
pNMN (nmol/L)	33	1.00	1.00–1.00	0.17
uNMN:Cr (nmol/mmol)	37	1	1–1	0.76
CVC invasion	71			
No (ref)	40			
Yes	31	1.00	0.72–1.39	0.99
Possible abdominal metastasis	71			
No (ref)	67			
Yes	4	0.83	0.46–1.51	0.55
Maximum tumor diameter	59	1.01	0.99–1.04	0.28
Thromboembolic disease	71			
No (ref)	57			
Yes	14	1.29	0.83–2.01	0.26
Treatment with chemotherapy agents	71			
No (ref)	60			
Yes	11	0.74	0.28–1.92	0.54
PBZ vs. PRZ	71			
PBZ (ref)	62			
PRZ	9	0.54	0.13–2.27	0.40

Abbreviations: ALP, alkaline phosphatase; ALT, alanine aminotransferase; FE, female entire; FN, female neutered; Group 1, adrenoreceptor‐antagonist treated; HCT, hematocrit; kg, kilograms; MN, male neutered; ME, male entire; NIBP, non‐invasive blood pressure; pNMN, plasma normetanephrine bpm, beats per minute; PLT, platelet count; PBZ, phenoxybenzamine; PRZ, prazosin; uNMN:Cr, urinary normetanephrine to creatinine ratio; WBC, white blood cell count.

^a^ Variables selected for multivariable analysis.

**TABLE 5:** Multivariable Cox proportional hazard analysis of risk of death (all‐cause) for Group 1.

	Hazard ratio	95% confidence interval	*p*
Vomiting (acute)			
No (ref)			
Yes	5.63	2.11–15.02	< 0.001
ALT (U/L)	1.00	1.00–1.00	0.02
Total protein (g/L)	1.05	1.00–1.09	0.04

Abbreviations: ALT, alanine aminotransferase; Group 1, adrenoreceptor‐antagonist treated.

Univariable results for Group 2 are presented in Table 6. Factors selected for inclusion in multivariable analysis were age at presentation, historical PU/PD and weight loss, the absence of physical examination abnormalities, heart rate, rectal temperature, surgical pretreatment with AAs, discontinuation of AAs before surgery, tumor extension beyond the diaphragm, and presence of postoperative complications, excluding cardiac arrest. Results of the final multivariable model are presented in Table 7.

**TABLE 6:** Univariable Cox proportional hazard analysis of risk of death (all‐cause) for Group 2.

	Number of cases	Hazard ratio	95% confidence interval	*p*
Age of presentation	123	1.26	1.04–1.53	0.02^a^
Sex	125			
FN (ref)	48			0.41
MN	51	2.23	0.60–2.50	0.57
FE	6	0.68	0.63–7.86	0.21
ME	20	1.01	0.25–1.90	0.47
Duration of clinical signs	122			
Incidental finding (ref)	32			0.83
Day of presentation	6	1.15	0.46–2.87	0.77
< 1 week	29	0.73	0.09–5.83	0.76
1–2 weeks	12	0.57	0.17–1.89	0.36
2–4 weeks	13	0.87	0.26–2.87	0.82
> 4 weeks	30	0.69	0.28–1.74	0.43
Exercise intolerance	125			
No (ref)	110			
Yes	15	2	0.82–4.87	0.13
Polyuria/polydipsia	125			
No (ref)	90			
Yes	35	0.44	0.19–1.01	0.05^a^
Vomiting (acute)	125			
No (ref)	99			
Yes	26	0.86	0.39–1.88	0.70
Weight loss	125			
No (ref)	107			
Yes	18	2.08	0.89–4.85	0.09^a^
Rectal temperature (°C)	85			
37.7–39.2 (ref)	71			0.02^a^
< 37.7	10	0.88	0.42–1.86	0.74
> 39.2	4	2.18	1.03–4.59	0.04
Heart rate (bpm)	102			
60–120 (ref)	78			
> 120	24	0.35	0.12–1.03	0.06^a^
Panting (presentation)	125			
No (ref)	90			
Yes	35	0.66	0.32–1.39	0.27
Respiratory rate (breaths/min)	125	0.99	0.95–1.04	0.71
Bodyweight (kg)	120	1.01	0.99–1.04	0.40
NIBP (mmHg)	68			
< 80	3			0.87
80–119	9	0.58	0.09–3.83	0.57
120–139	14	0.35	0.06–2.01	0.24
140–159	18	0.52	0.10–2.63	0.43
160–179	8	0.33	0.05–2.38	0.27
≥ 180	16	0.46	0.08–2.52	0.37
Abdominal distension	125			
No (ref)	111			
Yes	14	1.83	0.71–4.73	0.21
Abdominal pain	125			
No (ref)	103			
Yes	22	2.19	1.06–4.55	0.04
Heart murmur	125			
No (ref)	99			
Yes	26	1.04	0.46–2.37	
No physical examination abnormalities	125			
No (ref)	79			
Yes	46	0.51	0.25–1.01	0.05^a^
Anemia	110			
No (ref)	92			
Yes	18	1.61	0.70–3.71	0.27
WBC (×10^9^/L)	106	0.98	0.93–1.03	0.40
HCT (%)	113	0.98	0.94–1.03	0.44
PLT (×10^9^/L)	105	1	1.00–1.00	0.77
ALP (U/L)	107	1	1.00–1.00	0.80
ALT (U/L)	109	1	1.00–1.00	0.89
Bilirubin (mg/dL)	87	0.30	0.05–1.91	0.20
Chloride (mmol/L)	92	0.97	0.9–1.05	0.44
Creatinine (mg/dL)	112	1.10	0.53–2.26	0.80
Glucose (mg/dL)	86	1.00	0.99–1.01	0.97
Potassium (mmol/L)	103	0.96	0.55–1.67	0.88
Sodium (mmol/L)	103	0.97	0.90–1.04	0.41
Total calcium (mg/dL)	97	0.93	0.68–1.27	0.64
Total protein (g/L)	111	1.02	0.98–1.05	0.37
pNMN (nmol/L)	40	0.97	0.92–1.02	0.26
UNMN:Cr (nmol/mmol)	26	1	1.00–1.00	0.73
CVC invasion (imaging)	125			
No (ref)	73			
Yes	52	1.27	0.67–2.42	0.46
Possible abdominal metastasis	125			
No (ref)	110			
Yes	15	2.70	0.90–8.14	0.08^a^
Maximum tumor diameter	116	1.00	0.99–1.01	0.69
Thromboembolic disease	125			
No (ref)	103			
Yes	22	1.14	0.71–1.83	0.58
Treatment with chemotherapy agents	122			
No (ref)	113			
Yes	9	1.34	0.47–3.81	0.58
Adrenoreceptor‐antagonist pretreatment	117			
No (ref)	27			
Yes	90	0.44	0.13–0.89	0.02^a^
Pretreatment discontinued prior to surgery	77			
No (ref)	8			
Yes	78	0.35	0.10–1.21	0.10^a^
Tumor extension beyond the diaphragm	125			
No (ref)	121			
Yes	4	0.23	0.07–0.77	0.02^a^
Renal capsular invasion	125			
No (ref)	112			
Yes	13	1.08	0.42–2.77	0.88
Nephrectomy	125			
No (ref)	116			
Yes	9	1.12	0.39–3.17	0.84
Vascular invasion	125			
No (ref)	50			
Yes	75	1.49	0.78–2.82	0.22
Venotomy	125			
No (ref)	38			
Yes	87	1.23	0.64–2.38	0.53
Postoperative complications (any, excl. cardiac arrest)	115			
No (ref)	77			
Yes	38	3.08	1.61–5.89	< 0.001^a^
Mitotic figures (no./HPF)	87	0.94	0.85–1.04	0.25

Abbreviations: ALP, alkaline phosphatase; ALT, alanine aminotransferase; bpm, beats per minute; FE, female entire; FN, female neutered; Group 2, surgically treated; HCT, hematocrit; HPF, high‐power field; kg, kilograms; ME, male entire; MN, male neutered; PLT, platelet count; pNMN, plasma normetanephrine; UNMN:Cr, urinary normetanephrine to creatinine ratio; WBC, white blood cell count.

^a^ Variables selected for multivariable analysis.

**TABLE 7:** Multivariable Cox proportional hazard analysis of risk of death (all‐cause) for Group 2.

	Hazard ratio	95% confidence interval	*p*
Age of presentation	1.52	1.19–1.95	< 0.001
Weight loss			
No (ref)			
Yes	3.70	1.38–9.94	0.01
No physical examination abnormalities			
No (ref)			
Yes	0.30	0.12–0.75	0.01
Heart rate (bpm)			
60–120 (ref)			0.04
< 60	1.49	0.56–3.93	0.42
> 120	0.30	0.08–1.17	0.08
Rectal temperature (°C)			
37.7–39.2 (ref)			0.01
< 37.7	3.61	1.53–8.51	< 0.01
> 39.2	2.65	0.25–28.17	0.42
Postoperative complications (excl. cardiac arrest)			
No (ref)			
Yes	3.06	1.38–6.79	0.01

Abbreviations: bpm, beats per minute; Group 2, surgically treated.

## 4 Discussion

This study evaluates the clinical presentation and outcomes of dogs with PCC managed with AA without adrenalectomy and to compare these outcomes to those of dogs that were treated with adrenalectomy. The significantly shorter median OST of cases treated with AA without surgery, compared to those treated by adrenalectomy, along with adrenalectomy being a positive predictor of survival in multivariable analysis, supports adrenalectomy being the treatment of choice for dogs with PCC when overt metastatic disease has been ruled out, and surgery is feasible.

The literature regarding the outcome of dogs with non‐cortisol producing tumors left in situ is sparse. The median survival of dogs with invasive adrenal tumors where the endocrine functionality of the tumor was not described was 49 days, while the median survival of a cohort of dogs with noninvasive non‐cortisol producing adrenal tumors was 18 months [21, 25]. Neither of these studies specifically evaluated PCC and did not evaluate the use of AAs as a management approach. AAs have no known direct antitumor activity, but they might mitigate the catecholamine‐mediated effects of PCC, particularly hypertension, although no veterinary studies have specifically studied their use in dogs with PCC for this indication [26]. The relatively short median OST for AA‐treated dogs in this study brings into question the benefits of this treatment.

The factor associated with the highest hazard of shorter OST in Group 1 and 2 dogs, using COX, was the presence of weight loss. Cachexia is associated with an increased risk of death in small animal patients with cardiac disease and congestive heart failure and chronic kidney disease [27, 28, 29, 30]. Weight loss might also indicate increased disease severity or disease burden, although, conversely, the presence of metastatic disease was not retained as an independent predictor of death in our final model (Table 2). Increasing total protein was also a risk factor for death in the combined analysis of Group 1 and 2. A possible explanation is that the increased total protein concentration might reflect dehydration in these patients. The absence of physical examination abnormalities was associated with longer OST in both the combined (Group 1 and Group 2) and the Group 2 cases. This finding could be interpreted as those dogs who experience fewer endocrine consequences of their PCC having longer survival. Whether clinical signs could be used to predict tumor behavior, such as local recurrence or metastatic behavior, is yet to be determined.

Median survival after adrenalectomy in the present study of 927 days is consistent with prolonged survival times after adrenalectomy for PCC reported in the literature [2, 18, 31]. Previously reported risk factors for survival after adrenalectomy include the need for ureteronephrectomy, presence of postoperative pancreatitis or aspiration pneumonia, tumor size, presence of metastasis, and venous thrombosis, particularly where this extends beyond the diaphragm [20, 32, 33, 34]. The development of postoperative complications carried a three times greater hazard of death among Group 2 dogs in this study. Individual complications were not included in hazard analysis due to the low frequency with which these were recorded (Table S7). Additionally, the collection of this data was based on defined categories from initial data gathered by the primary authors (MMES, CJS, RG), meaning that other complications might have been overlooked during data entry. Neither vascular invasion nor the need for a nephrectomy was significant in univariable analysis of this study. Vascular invasion beyond the diaphragm was a significant predictor of reduced survival in univariable analysis (Table 6) but was not retained in the final model. Other differences in risk factors might reflect differences in study design and study groups, given that surgical studies are more often designed to assess perioperative mortality or combine analysis of both cortical and medullary tumors. Although dogs experiencing perioperative mortality were not excluded from survival analysis and this might have affected results, perioperative mortality in this study was relatively low with only 6.3% (*n* = 8) of surgical cases dying before discharge.

Preoperative treatment with PBZ in dogs with PCC was common in this study, consistent with recommendations in veterinary review articles and textbook chapters [3, 4, 5, 26]. This is largely based on a single retrospective study reporting a bivariate statistical difference between dogs pretreated with PBZ and those without pretreatment [17]. This previous study was not able to consider confounding variables through multivariable analysis and might have been affected by temporal factors. Furthermore, subsequent studies have not replicated the outcome that PBZ is related to perioperative survival and patients can have good outcomes without preoperative treatment [18, 19, 35]. Although the present study did not specifically investigate risk factors for perioperative mortality and small numbers of perioperative deaths might have impacted the statistical comparison of this specific factor, survival to discharge among dogs undergoing adrenalectomy receiving AA pretreatment was significantly greater than those that were not pretreated when analyzed by Fisher's exact test. Pretreatment with AA was also significantly protective in univariable CPH modeling for dogs who underwent adrenalectomy but failed to retain significance in multivariable analysis. Veterinary literature suggests PBZ pretreatment might not impact intraoperative blood pressure and can be associated with more episodes of hypertension and arrhythmias intraoperatively [18, 36]. Therefore, it is possible that the reported survival benefit of PBZ in this and the aforementioned study could be due to factors that are not directly related to effects on intraoperative cardiovascular variables. Furthermore, given the small number of dogs with perioperative mortality in this study, it was considered inappropriate to make comparisons between those that were pretreated versus non‐pretreated, so it would be prudent not to draw strong conclusions on whether PBZ pretreatment should or should not be used based on these data.

Surgical death rates in this study might have been affected by low numbers of cases with more severe, acute presentations, compared to the number of cases with less acute presentations, given that the median time from diagnosis to surgery was 32 (range 0–694) days and only 4.7% (*n* = 6, 2 cases with missing data) cases had surgery on the day of presentation, with all these cases surviving to discharge. A recent study has demonstrated a large difference in case fatality rates for emergent versus elective adrenalectomy (50% vs. 5.7%, respectively), which highlights this factor as a potential confounder in our study group [37]. Although a subsequent study suggested fatality rates in more urgent presentations can be mitigated if surgery can be delayed [38]. Furthermore, although it was possible to collect objective individual data on cases in this study, it is not possible to accurately classify the severity of presenting signs retrospectively and therefore not possible to determine the effect of the severity of cardiovascular compromise on death rates in dogs within our study group.

A potential limitation of this study is that there is no single method that confirms the presence of a PCC when histopathology has not been performed, which is inherent to Group 1 cases in this study. Our study used a pNMN of > 5.52 mmol/L and uNMN:Cr of greater than 4 times the upper reference interval for uNMN:Cr, or in the case of laboratories without a reference interval greater than 400 nmol/mmol based on available literature in dogs [6, 8, 10]. A more recent study has shown lower cutoffs for pNMN could provide comparable sensitivity and specificity, although this might partly be due to differences in laboratory technique [7]. Furthermore, measurement of metanephrines in either urine or plasma can be affected to some degree by sample handling [39]. Given its retrospective nature, it was not possible to confirm the specific handling of all samples. Our chosen cutoffs were selected to increase our confidence that only genuine PCC cases were included, but it is possible we inadvertently excluded true cases that had less marked increases in metanephrines, or only intermittent secretion of catecholamines and that our findings might not be applicable to that cohort. Additionally, the decision to exclude cases based on medications that could interfere with metanephrine assessment was intended to further prevent inadvertent inclusion of non‐PCC cases; although equally, it might have led to the exclusion of some true PCC cases. Finally, an important differential diagnosis for functional adrenal tumors is canine Cushing's syndrome (CCS). Although the cutoffs used in this study for uNMN:Cr and pNMN have been shown to differentiate dogs with PCC from those with CCS, it was not a requirement that all cases had to have an LDDST performed to include them in our dataset [6, 8].

Age at presentation was statistically significant when evaluating for treatment selection bias. However, the actual difference was relatively small and was therefore not expected to have a large impact on subsequent analyses or conclusions that can be drawn from survival analysis. Although biases in treatment selection were noted at two centers, the number of cases from both centers was relatively small, and they also reflect the real‐world situations and decisions that are made in practice. It was interesting to note that the possibility of abdominal metastatic disease on diagnostic imaging did not reach significance in the analysis of reasons for treatment selection and was not a risk factor for reduced survival in any of our CPH models. This variable did not require confirmation of metastatic disease as part of the study design and might have captured cases with, for example, splenic or hepatic nodules that would not necessarily indicate a neoplastic process. Furthermore, the number of cases with possible thoracic metastatic disease was low (Table S5) which precluded its use in our statistical models. This is a limitation of this retrospective study design and anonymization of case data; therefore, we would caution against concluding that metastatic disease is, or is not, a prognostic factor in these cases.

Other limitations to the study are inherent to its multicenter and retrospective design. It was not possible, from these data, to determine if all the cases within the AA group were intended to only have AA treatment versus receiving AA treatment while awaiting surgery. If these cases were to die before surgery, it is possible that they might have been recorded in the AA group, rather than surgical, thus biasing survival times in favor of surgical treatment. Retrospective data entry requires accurate clinical records, and there is the possibility that some data were not captured at the point of presentation and therefore not recorded in this retrospective analysis. Although data entry was standardized using an online data capture system, some degree of subjectivity is inherent to the questions asked of the individual entering data, and although attempts were made to minimize missing data through the review of anonymized data by the primary author (MS), access to case records was not always possible, and it is possible that some data were not accurate or was entered incorrectly. Inaccurate recall or interpretation of clinical records might account for some data, such as the higher frequency of renal capsular invasion reported during adrenalectomy in Group 2 dogs than elsewhere in veterinary literature. Although missing data were accounted for in statistical modeling, and censoring of cases due to missing data is assumed to be random, the outcomes of the models and the interpretation of results should bear this in mind. Equally, AA dose protocols and adherence to these by owners is not possible to confirm in a retrospective study of this nature. Type 1 statistical error might also be a reason for some variables being significant in our models. Survival analysis of Group 3 was not performed because a large proportion of this group had limited follow‐up and lack of treatment was not considered an active management decision in this study, resulting in a lack of date of intention not to treat, along with a follow‐up date. Therefore, further study with a standardized follow‐up protocol for cases that receive neither AA nor surgery could be considered. Finally, given that this study used overall survival rather than disease‐specific survival, this limits the ability to conclude that outcomes in this study are purely due to PCC or the PCC‐directed treatments alone. For example, there might be reasons behind the treatment of cases with AA alone, as opposed to surgical treatment, that cannot be fully elucidated from these retrospective data, and future prospective study that includes disease‐specific survival would be best placed to improve understanding of PCC and PCC treatment's direct effects on survival.

## 5 Conclusion

This study reports characteristics and survival in dogs with PCC treated with AA alone, alongside comparing survival to those undergoing adrenalectomy. Despite the inherent limitations of a study of this kind, this data supports that adrenalectomy is the treatment associated with the longest median survival time. The results of this study are likely to be of use to veterinarians when counseling owners on likely outcomes in cases of dogs with PCC.

## Disclosure

The authors declare no off‐label use of antimicrobials.

## Ethics Statement

This study was approved by the Social Science and Ethical Review Board at the Royal Veterinary College (URN SR2020‐0244). The authors declare human ethics approval was not needed.

## Conflicts of Interest

The authors declare no conflicts of interest.

## Supporting information


**Data S1:** Supporting information.
