# Supplementary material for: A Multicenter, Retrospective Analysis of Long‐Term Survival in 255 Dogs With Pheochromocytoma Treated With Alpha‐Adrenoreceptor Antagonists or Surgery (2010–2021)
Source: J Vet Intern Med. 2025 Sep 1;39(5):e70220. doi: 10.1111/jvim.70220 (PMC12402397; doi:10.1111/jvim.70220)
Supplement: Supplementary file 1 — Data S1: Supporting information. [file JVIM-39-e70220-s001.docx]

Supplemental Information

Table of Contents

[Supplemental Table S1: Number of cases from each contributing center 3](#_Toc195511322)

[Supplemental Table S2: Demographic characteristics of cases of canine pheochromocytoma. 5](#_Toc195511323)

[Supplemental Figure S1: Breed Distribution 8](#_Toc195511324)

[Supplemental Figure S2: Duration of clinical signs prior to presentation in cases of canine pheochromocytoma. 9](#_Toc195511325)

[Supplementary Figure S3: Historical and Clinical Signs in Cases of Canine Pheochromocytoma 11](#_Toc195511326)

[Supplemental Table S3: Clinicopathologic Data for Cases of Canine Pheochromocytoma 12](#_Toc195511327)

[Supplemental Table S4: Frequency of cases of canine pheochromocytoma blood pressure measurements 19](#_Toc195511328)

[Supplemental Table S5: Imaging Characteristics of Canine Pheochromocytoma Cases 20](#_Toc195511329)

[Histopathologic Findings in Cases of Canine Pheochromocytoma 24](#_Toc195511330)

[Supplemental Table S6: Surgical Pre-Treatment Variables 25](#_Toc195511331)

[Supplemental Table S7: Post Operative Complications 28](#_Toc195511332)

[Outcomes of Group 3 31](#_Toc195511333)

[Supplemental Figure S4: Kaplan-Meier estimate of follow up time for group 3 (no treatment) cases of canine PCC. 32](#_Toc195511334)

[Chemotherapy Details 33](#_Toc195511335)

[Supplemental Table S8: Causes of Death 34](#_Toc195511336)

## Supplemental Table S1: Number of cases from each contributing center

| Center | Total Number of Cases | Group 1 Cases | Group 2 Cases | Group 3 Cases |
| --- | --- | --- | --- | --- |
| AM, UK | 17 (6.7%) | 6 (8.0%) | 3 (2.3%) | 8 (15.4%) |
| ANV, ES | 3 (1.2%) | 2 (2.7%) | 1 (0.8%) | 0 |
| DVS, UK | 23 (9.0%) | 4 (5.3%) | 14 (10.9%) | 5 (9.6%) |
| DWR, UK | 6 (2.4%) | 3 (4.0%) | 1 (0.8%) | 2 (3.8%) |
| ECT, UK | 2 (0.8%) | 1 (1.3%) | 1 (0.8%) | 0 |
| EVC, FR | 3 (1.2%) | 0 | 1 (0.8%) | 2 (3.8%) |
| HVS, UK | 2 (0.8%) | 1 (1.3%) | 0 | 1 (1.9%) |
| LP, UK | 2 (0.8%) | 0 | 2 (1.6%) | 0 |
| NDSR, UK | 12 (4.7%) | 5 (6.7%) | 7 (5.5%) | 0 |
| NVA, FR | 2 (0.8%) | 0 | 2 (1.6%) | 0 |
| PVR, UK | 4 (1.6%) | 1 (1.3%) | 1 (0.8%) | 2 (3.8%) |
| QMHA, UK | 43 (16.9%) | 11 (14.7%) | 24 (18.8%) | 8 (15.4%) |
| UoA, FR | 14 (5.5%) | 3 (4.0%) | 3 (2.3%) | 8 (15.4%) |
| UoBer, CH | 7 (2.7%) | 3 (4.0%) | 4 (3.1%) | 0 |
| UoBol, IT | 8 (3.1%) | 0 | 8 (6.3%) | 0 |
| UoBr, UK | 7 (2.7%) | 1 (1.3%) | 4 (3.1%) | 2 (3.8%) |
| UoC, UK | 6 (2.4%) | 0 | 5 (3.9%) | 1 (1.9%) |
| UoD, IE | 3 (1.2%) | 0 | 2 (1.6%) | 1 (1.9%) |
| UoE, UK | 9 (3.5%) | 5 (6.7%) | 2 (1.6%) | 2 (3.8%) |
| UoL, BE | 4 (1.6%) | 1 (1.3%) | 3 (2.3%) | 0 |
| UoLis, PT | 3 (1.2%) | 2 (2.7%) | 0 | 1 (1.9%) |
| UoLiv, UK | 5 (2.0%) | 1 (1.3%) | 4 (3.1%) | 0 |
| UoLP, DE | 5 (2.0%) | 3 (4.0%) | 2 (1.6%) | 0 |
| UoZ, CH | 14 (5.5%) | 9 (12.0%) | 3 (2.3%) | 2 (3.8%) |
| UU, NL | 38 (14.9%) | 11 (14.7%) | 20 (15.6%) | 7 (13.5%) |
| WVS, UK | 13 (5.1%) | 2 (2.7%) | 11 (8.6%) | 0 |
| Total | **255** | **75** | **128** | **52** |

Percentages indicate the percentage of cases within the corresponding group from each center. AM, Anderson Moores Veterinary Specialists; ANV, Anicura Hospital Valencia; DVS, Davies Veterinary Specialists; ECT, Eastcott Veterinary Referrals; EVC, Evolia Veterinary Clinic; HVS, Highcroft Veterinary Specialists; LP, Lumbry Park Veterinary Specialists; NDSR, North Downs Specialist Referrals; NVA, Nordvet Anicura France; PVR, Pride Veterinary Referrals; QMHA, Queen Mother Hospital for Animals, Royal Veterinary College; UoA, Alfort School of Veterinary Medicine; UoBer, University of Bern; UoBol, University of Bologna; UoBr, University of Bristol; UoC, Queen’s Veterinary School Teaching Hospital, University of Cambridge; UoD, University of Dublin; UoE, The Royal (Dick) School of Veterinary Studies, University of Edinburgh; UoL, University of Leige; UoLis, University of Lisbon; UoLiv, Small Animal Teaching Hospital, University of Liverpool; UoLP, University of Leipzig; UoZ, University of Zurich; UU, Utrecht University; WVS, Willows Veterinary Specialists. UK, United Kingdom; ES, Spain; FR, France; CH, Switzerland;IT, Italy; IE, Republic of Ireland; BE, Belgium; PT, Portugal; DE, Germany; NL, Netherlands.

## Supplemental Table S2: Demographic characteristics of cases of canine pheochromocytoma.

| Variable | Overall (n = 255) | Medical Treatment (n = 75)  *Group 1* | Surgical (n = 128)  *Group 2* | Untreated (n = 52)  *Group 3* |
| --- | --- | --- | --- | --- |
| Sex  FN  MN  FE  ME | 92 (36.1%) 101 (39.6%)  14 (5.5%)  48 (18.8%) | 23 (30.7%)  29 (38.7%)  4 (5.3%)  19 (25.3%) | 50 (39.1%)  52 (40.6%)  6 (4.7%)  (15.6%) | 19 (36.5%)  20 (38.5%)  4 (7.7%)  9 (17.3%) |
| Weight (kg^a^) | n = 245  14.6 (3.6 – 64.0) | n = 74  12.5 (3.6 – 64.0) | n = 123 (3.9%)  16.0 (4.3 – 53.0) | n = 48  20 (3.8 – 63.5) |
| Body Condition Score (1 – 9) | n = 178  5 (2 – 9) | n = 59  5 (2 – 9) | n = 88  5 (2 – 8) | n = 31  5 (3 – 8) |
| Age at presentation (years) | n = 251  11 (1 – 16) | n = 73  11 (5 – 15) | n = 126  10 (4 – 14) | n = 52 11 (1 – 16) |
| Rectal Temperature  (Celsius) | n = 177  38.4 (33.8 – 40.6) | n = 52 38.3 (35.9 – 39.8) | n = 86  38.4 (36.5 – 39.6) | n = 39  38.5 (33.8 – 40.6) |
| Heart Rate  (beats/minute) | n = 213  110 (50 – 260) | n = 66  111 (72 – 240) | n = 104  110 (66 – 260) | n = 43  108 (50 – 220) |
| Respiratory Rate  (breaths/minute) | n = 124  28 (12 – 100) | n = 42  29 (18 – 80) | n = 58  24 (12 – 60) | n = 26  32 (16 – 100) |
| Number of co-morbidities | 0 = 224 (87.8%)  1 = 25 (9.8%)  2 = 5 (2.0%)  3 = 1 (0.4%) | 0 = 68 (90.7%)  1 = 7 (9.3%)  2 = 0  3 = 0 | 0 = 121 (94.5%)  1 = 6 (4.7%)  2 = 1 (0.8%)  3 = 0 | 0 = 35 (67.3%)  1 = 12 (23.1%)  2 = 4 (7.7%)  3 = 1 (1.9%) |

Categorical data is presented as number (percentage) and continuous data as mean/median (standard deviation/range). Percentages of categorical data are presented as proportions of the group indicated, and of variables with missing data, as a percentage of the cases of which data is present. ^a^kilograms. *FN, female neutered; MN, male neutered; FE, female entire; ME, male entire.*


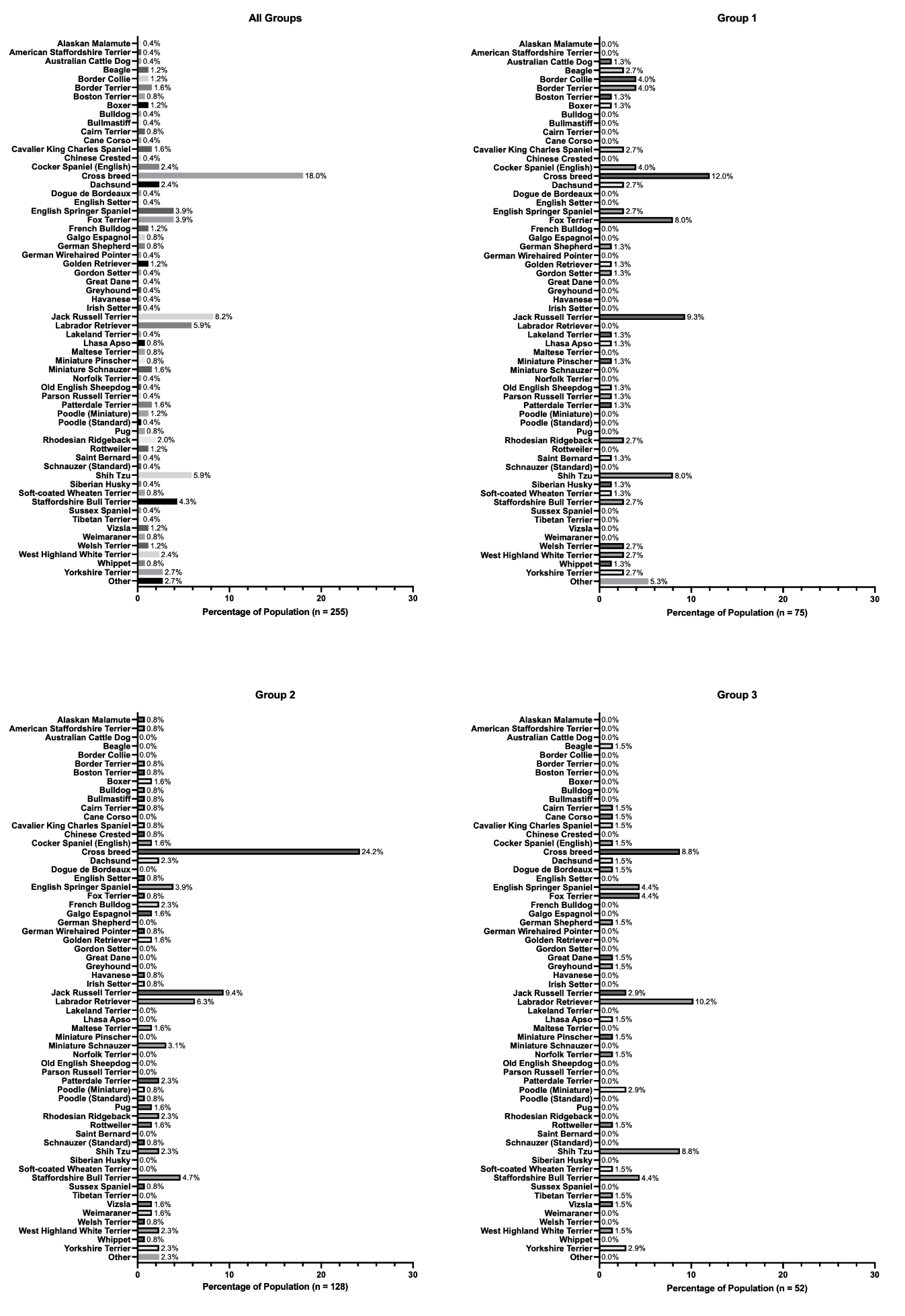


Supplemental Figure S1: Breed Distribution *Percentage of breeds by treatment group for canine pheochromocytoma cases – top left to bottom right: All cases, group 1, group 2 group 3*


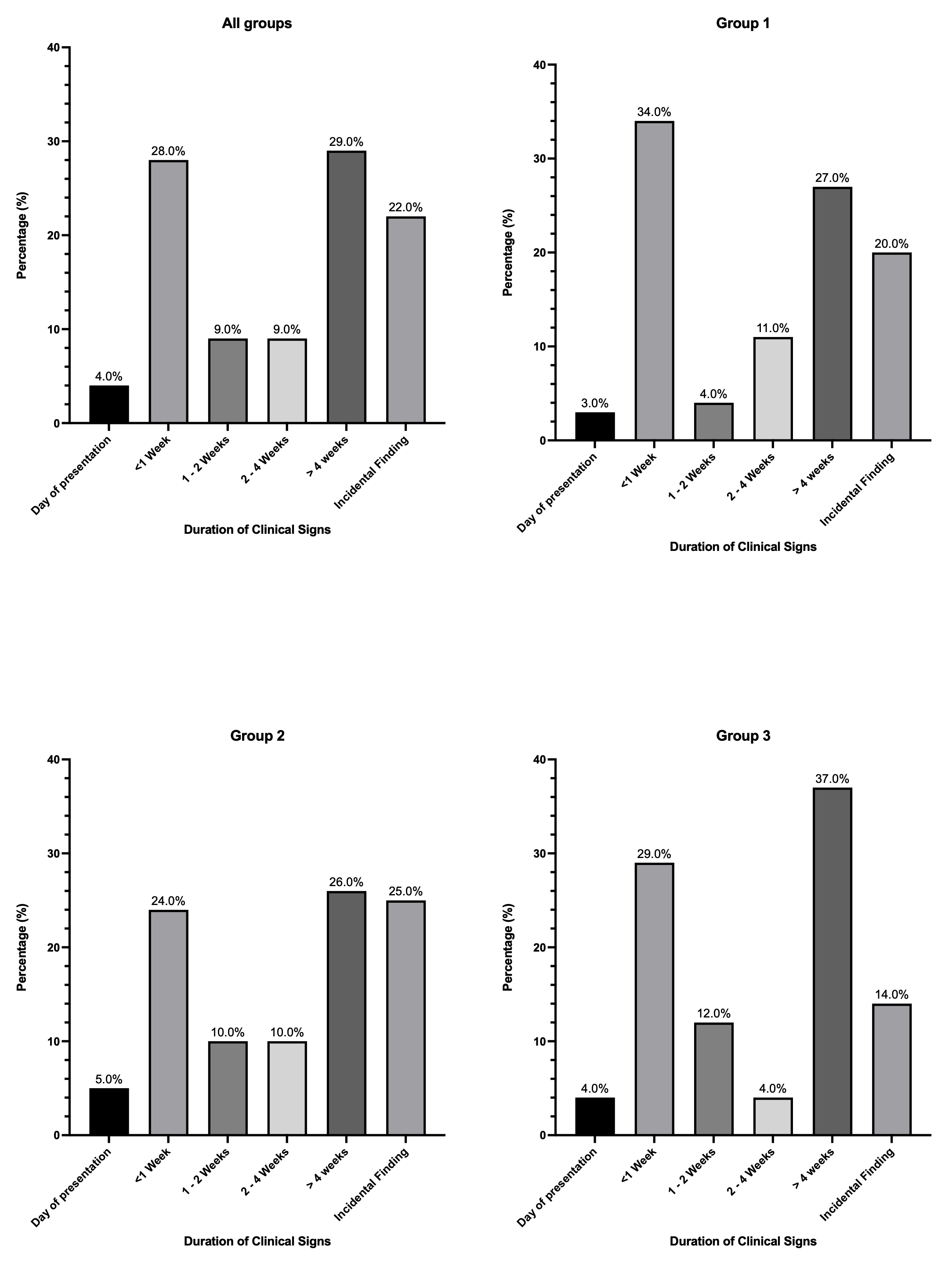


Supplemental Figure S2: Duration of clinical signs prior to presentation in cases of canine pheochromocytoma. *Top left to bottom right: All cases, group 1, group 2 group. The distribution of cases with the indicated duration of clinical signs are presented as a percentage of the number of cases in which information was recorded. Missing values in each group were: 14/255 (5.5%) all cases, 6/75 (8%) group 1, 3/128 (2.3%) group 2 and 5/52 (9.6%) group*


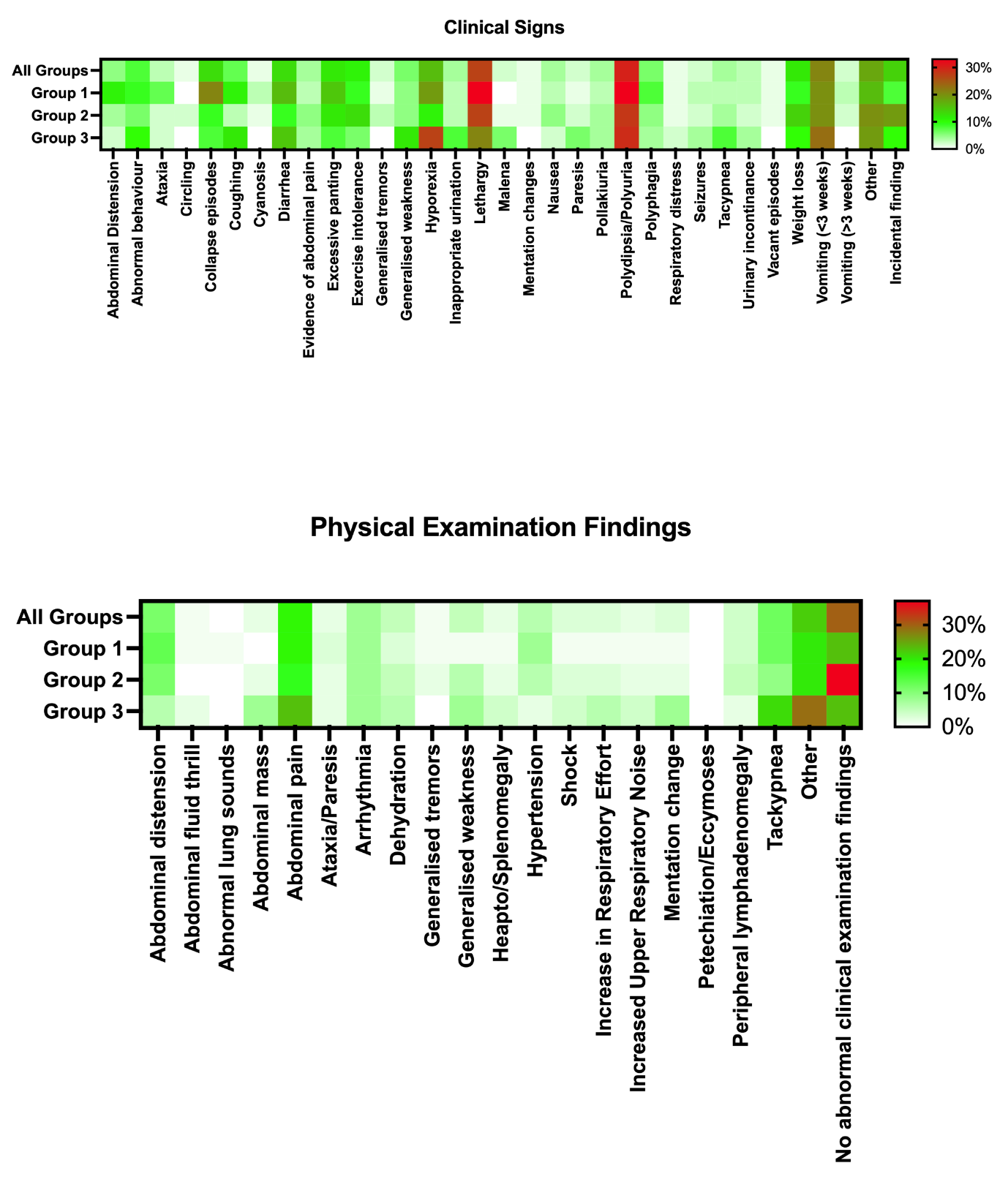


Supplementary Figure S3: Historical and Clinical Signs in Cases of Canine Pheochromocytoma

Heatmaps indicating the percentage of each group presenting with a given historical or physical examination finding.

## Supplemental Table S3: Clinicopathologic Data for Cases of Canine Pheochromocytoma

| Variable | Overall (n = 255) | Medical Treatment (n = 75)  *Group 1* | Surgical (n = 128)  *Group 2* | Untreated (n = 52)  *Group 3* |
| --- | --- | --- | --- | --- |
| White blood cell count  (x10^9/L) | n = 213  9.7 (2.5 – 49.8) | n = 62  9.4 (2.5 – 38.1) | n = 109  9.5 (3.7 – 42.5) | n = 42  10.5 (4.7 – 49.8) |
| Neutrophil toxic change | n = 208  Yes = 19 (9.1%)  No = 189 (90.9%) | n = 62  Yes = 6 (9.7%)  No = 56 (90.3%) | n = 106  Yes = 6 (5.7%)  No = 100 (94.3%) | n = 40  Yes = 7 (17.5%)  No = 33 (82.5%) |
| Band neutrophilia | n = 212  No = 193 (91.0%)  Yes = 19 (9.0%) | n = 62  Yes = 6 (9.7%)  No = 56 (90.3%) | n = 109  Yes = 7 (6.4%)  No = 102 (93.6%) | n = 41  Yes = 6 (14.6%)  No = 35 (67.3%) |
| PCV/HCT^1^  (%) | n = 255  44.4 (12.0 – 67.3) | n = 66  44.0 (19.0 – 62.0) | n = 106  45.3 (12.0 – 67.3) | n = 43  43.0 (19.7 – 56.0) |
| Reticulocyte Count  (x10^9/L) | n = 123  57.1 (0.0 – 52200.0) | n = 40  80.9 (15.3 – 40600.0) | n = 61  450 (0.0 – 32200.0) | n = 22  48.7 (15.5 – 52200.0) |
| Anemia | n = 216  Yes = 39 (18.1%)  No = 177 (69.4%) | n = 63  Yes = 14 (22.2%)  No = 49 (77.8%) | n = 112  Yes = 18 (16.1%)  No = 94 (83.9%) | n = 41  Yes = 7 (17.1%)  No = 34 (82.9%) |
| Platelet Count  (x10^9/L) | n = 205  335 (32 – 977) | n = 58  340 (32 – 716) | n = 108  320 (56 – 977) | n = 39  359 (75 – 862) |
| Glucose  (mg/dL)  [mmol/L] | n = 181  102.6 (39.6 – 347.4)  [5.7 (2.2 – 19.3)] | n = 55  105.0 (55.8 – 230.4)  [6.0 (3.1 – 12.8)] | n = 88  100.8 (55.8 – 347.4)  [5.6 (3.1 – 19.3)] | n = 38  97.2 (39.6 – 307.8)  [5.4 (2.2 – 17.1)] |
| Sodium  (mmol/L) | n = 218  149.0 (137.0 – 172.0) | n = 68  149.0 (140.0 – 172.0) | n = 106  148.0 (137.0 – 159.0) | n = 44  149.0 (141.0 – 162.0) |
| Chloride  (mmol/L) | n = 189  112.0 (98.0 – 135.0) | n = 56  112.1 (98.0 – 135.0) | n = 95  112.0 (99.0 – 129.0) | n = 38  112.5 (103.6 – 122.0) |
| Potassium  (mmol/L) | n = 220  4.5 (2.3 – 8.2) | n = 69  4.5 (2.3 – 5.8) | n = 106  4.5 (3.2 – 8.2) | n = 45  4.5 (3.0 – 6.1) |
| Total Calcium  (mg/dL)  [mmol/L] | n = 192  10.4 (6.0 – 14.8)  [2.6 (1.5 – 3.7)] | n = 58  10.8 (6.8 – 14.8)  [2.7 (1.7 – 3.7)] | n = 99  10.4 (6.0 – 12.8)  [2.6 (1.5 – 3.2)] | n = 35  10.0 (7.2 – 12.4)  [2.5 (1.8 – 3.1)] |
| Ionised Calcium  (mg/dL)  [mmol/L] | n = 57  5.12 (2.00 – 5.60)  [1.28 (0.50 – 1.70)] | n = 14  5.20 (2.00 – 5.60)  [1.30 (0.50 – 1.40)] | n = 33  5.12 (2.00 – 6.80)  [1.28 (0.50 – 1.70)] | n = 10  5.00 (4.00 – 5.60)  [1.25 (1.00 – 1.40)] |
| Total Protein  (g/L) | n = 223  65.0 (35.8 – 93.1) | n = 63  64.1 (37.0 – 84.0) | n = 114  65.7 (43.2 – 93.1) | n = 46  65.2 (35.8 – 84.0) |
| Albumin  (g/L) | n = 214  32.0 (19.0 – 47.0) | n = 62  32.7 (19.0 – 47.0) | n = 112  32.9 (19.4 – 47.0) | n = 40  31.2 (22.5 – 42.0) |
| Globulin  (g/L) | n = 182  33 (10.7 – 53.4) | n = 48  34.0 (17.0 – 50.0) | n = 96  32.5 (19.0 – 53.4) | n = 38  33.9 (10.7 – 52.0) |
| ALT (U/L) | n = 219  79.0 (10.0 – 37310.0) | n = 63  80.0 (13.0 – 1162.0) | n = 112  79.0 (19.0 – 477.0) | n = 46  76.2 (10.0 – 32310.0) |
| ALP (U/L) | n = 214  183.5 (2.4 – 4004.0) | n = 61  145.0 (16.0 – 4004.0) | n = 110  192.5 (2.4 – 3081.0) | n = 43  161.0 (29.0 – 1805.0) |
| Total bilirubin  (mg/dL)  [μmol/L] | n = 172  0.17 (0.00 – 2.29)  [3.0 (0.0 – 39.4)] | n = 50  0.16 (0.00 – 0.41)  [2.8 (0.0 – 7.0)] | n = 89  0.18 (0.00 – 2.29)  [3.1 (0.0 – 39.4)] | n = 33  0.18 (0.00 – 1.82)  [3.1 (0.0 – 31.3)] |
| Cholesterol  (mg/dL)  [mmol/L] | n = 173  255.4 (0.0 – 650.2)  [6.6 (0.0 – 16.8)] | n = 48  255.4 (0.0 – 445.1)  [6.6 (0.0 – 11.5)] | n = 90  259.3 (112.2 – 650.2)  [6.7 (2.9 – 16.8)] | n = 35  239.9 (123.8 – 472.1)  [6.2 (3.2 – 12.2)] |
| Urea  (mg/dL)  [mmol/L] | n = 228  17.64 (2.24 – 190.40)  [6.3 (0.8 – 68.0)] | n = 70  17.92 (5.60 – 190.40)  [6.4 (2.0 – 68.0)] | n = 113  17.08 (2.24 – 156.24)  [6.1 (0.8 – 55.8)] | n = 45  15.68 (3.08 – 104.16)  [5.6 (1.1 – 37.2)] |
| Creatinine  (mg/dL)  [μmol/L] | n = 232  0.91 (0.01 – 8.15)  [83.0 (1.0 – 741.0)] | n = 72  0.092 (0.01 – 8.15)  [83.5 (1.0 – 741.0)] | n = 115  0.88 (0.41 – 3.80)  [80.0 (37.0 – 345.0)] | n = 45  1.09 (0.58 – 4.99)  [99.0 (53.0 – 454.0)] |
| USG^2^ | n = 160  1.022 (1.001 – 1.050) | n = 56  1.021 (1.006 – 1.050) | n = 75  1.025 (1.001 – 1.050) | n = 29  1.019 (1.004 – 1.050) |
| UPC^3^ | n = 116  0.57 (0.0 – 18.0) | n = 34  0.895 (0.0 – 10.0) | n = 59  0.5 (0.1 – 18.0) | n = 23  0.6 (0.1 – 8.6) |

Categorical data is presented as number (percentage) and continuous data as mean/median (standard deviation/range). Percentages of categorical data are presented as proportions of the group indicated, and of variables with missing data, as a percentage of the cases of which data is present. ^1^Packed cell volume/haematocrit, ^2^urine specific gravity, ^3^urine protein:creatinine.

## Supplemental Table S4: Frequency of cases of canine pheochromocytoma blood pressure measurements

|  | Overall (n = 255)  Number (%) | Group 1 (n = 75) | Group 2 (n = 128) | Group 3 (n = 52) |
| --- | --- | --- | --- | --- |
| <120 mmHg | 22 (17.4%) | 6 (15.8%) | 12 (17.4%) | 4 (21.0%) |
| 120 – 139 mmHg | 27 (21.4%) | 6 (15.8%) | 14 (20.3%) | 7 (36.8%) |
| 140 – 159 mmHg | 28 (22.2%) | 8 (21.1%) | 18 (26.1%) | 2 (10.5%) |
| 160 – 179 mmHg | 22 (17.5%) | 10 (26.3%) | 9 (13.0%) | 3 (15.8%) |
| ≥180 mmHG | 27 (21.4%) | 8 (21.1%) | 16 (23.2%) | 3 (15.8%) |

Based on minimum pre-tx value. Percentage represents frequency of blood pressure measurements across cases in which a measurement was available.

## Supplemental Table S5: Imaging Characteristics of Canine Pheochromocytoma Cases

|  | All Groups (n = 255)  Number (%) | Group 1  (n = 75) | Group 2  (n = 128) | Group 3  (n = 52) |
| --- | --- | --- | --- | --- |
| Imaging Performed |  |  |  |  |
| Thoracic radiographs | 83 (32.5%) | 29 (38.7%) | 38 (29.7%) | 16 (36.5%) |
| Abdominal radiographs | 15 (5.9%) | 4 (5.3%) | 8 (6.3%) | 3 (5.8%) |
| Abdominal ultrasound | 163 (63.9%) | 51 (68.0%) | 76 (59.4%) | 36 (69.2%) |
| Thoracic Computed Tomography | 138 (54.1%) | 34 (45.3%) | 85 (66.4%) | 19 (36.5%) |
| Abdominal Computed Tomography | 157 (61.6%) | 35 (46.7%) | 103 (80.5%) | 19 (36.5%) |
| Magnetic Resonance Imaging | 8 (3.1%) | 4 (5.3%) | 1 (0.8%) | 3 (5.8%) |
|  |  |  |  |  |
| Affected adrenal | Unknown = 9 (3.5%) | Unknown = 3 (4.0%) | Unknown = 2 (1.6%) | Unknown = 4 (7.75) |
| Right Adrenal | 99 (40.2%) | 25 (34.7%) | 59 (46.8%) | 15 (31.3%) |
| Left Adrenal | 136 (55.3%) | 40 (55.6%) | 66 (52.4%) | 30 (62.5%) |
| Bilateral | 11 (4.5%) | 7 (9.7%) | 1 (0.8%) | 3 (6.3%) |
|  |  |  |  |  |
| Contralateral Adrenal Gland description | Unknown = 47 (18.4%) | Unknown = 14 (18.7%) | Unknown = 21 (16.4%) | Unknown = 12 (23.1%) |
| Increased size | 26 (12.5%) | 9 (14.8%) | 11 (10.3%) | 6 (1.5%) |
| Decreased size | 12 (5.8%) | 2 (3.3%) | 9 (8.4%) | 1 (2.5%) |
| Mass | 7 (3.4%) | 1 (1.6%) | 3 (2.8%) | 3 (7.5%) |
| Normal size | 163 (78.4%) | 49 (80.3%) | 84 (78.5%) | 30 (75.0%) |
|  |  |  |  |  |
| Other Imaging findings |  |  |  |  |
| Possible thoracic metastasis | 6 (2.4%) | 1 (1.3%) | 3 (2.3%) | 2 (3.8%) |
| Possible abdominal metastasis | 33 (12.9%) | 5 (6.7%) | 17 (13.3%) | 11 (21.2%) |
| Thromboembolic disease (thoracic) | 9 (3.5%) | 3 (4%) | 5 (3.9%) | 1 (1.9%) |
| Thromboembolic disease (abdominal) | 27 (10.6%) | 8 (10.7%) | 16 (12.5%) | 13 (25.0%) |
| Maximum tumour diameter (mm) | Unknown = 26 (10.2%)  28.0 (1.8 – 278.0) | Unknown = 12 (16.0%)  26.0 (5.0 – 88.0) | Unknown = 9 (7.8%)  28.0 (2.5 – 278.0) | Unknown = 5 (9.6%)  27.0 (1.8 – 150.0) |
| CVC Invasion | 114 (44.7%) | 31 (41.3%) | 55 (43.0%) | 28 (53.8%) |

Categorical data is presented as number (percentage) and continuous data as mean/median (standard deviation/range). Percentages of categorical data are presented as proportions of the group indicated, and of variables with missing data, as a percentage of the cases of which data is present.

## Histopathologic Findings in Cases of Canine Pheochromocytoma

A total of 149/255 (58.3%) cases had adrenal histopathology performed including 63/149 (42.3%) cases performed on the right adrenal gland only and 75/149 (50.3%) performed on the left gland only. 7/149 (4.7%) cases were noted to have histopathology in both glands and the side of histopathology was unknown in 4 cases. Histopathology was performed in only 5/75 (6.7%) cases that had medical treatment, 127/128 (99.2%) cases that had surgical treatment and 17/35 (48.6%) cases that had neither AA or surgical treatment.

Histopathologic diagnosis in cases that had single glands analysed all had a diagnosis of PCC other than one case with a diagnosis of a combined right sided PCC, cortical adenoma and myelolipoma.

In the 7 cases where both glands where analysed, three cases had bilateral PCC, two cases had a left sided PCC with right sided cortical hypertrophy, one case had a right sided PCC and right sided adrenocortical adenoma, in this case, interestingly, the left gland showed evidence of medullary, but not cortical atrophy. Another case had a left sided PCC but normal right adrenal gland.

## Supplemental Table S6: Surgical Pre-Treatment Variables

| Surgical Pre-Treatment Variable | Median (range) | Number | Percentage (%) |
| --- | --- | --- | --- |
| Cases receiving pre-treatment |  | 92 | 71.9 |
| PBZ |  | 81 | 88.0 |
| PRZ |  | 11 | 12.0 |
| Duration of treatment  < 1 week  1 – 2 weeks  2 – 4 weeks  >4 weeks |  | Unknown = 4  11  17  44  16 | 4.3  12.5  19.3  50  18.2 |
| PBZ: starting dose | 0.3 (0.2 – 3.0) mg/kg | Unknown = 5 | 5.4 |
| Treatment Frequency (PBZ)  SID  BID  TID |  | Unknown = 4  4  73  1 | 4.9  4.8  90.1  1.2 |
| PBZ discontinued prior to surgery |  | 8 | 9.8 |
| PBZ: dose escalations |  | 14 | 15.2 |
| PRZ: starting dose | 0.17 (0.1 – 1.0) mg/kg |  |  |
| Treatment Frequency (PRZ)  SID  BID  TID |  | 2  7  2 | 18.2  63.6  18.2 |
| PRZ: discontinued prior to surgery |  | 1 | 9.1% |
| PRZ: dose escalations |  | 2 | 18.2% |

## Supplemental Table S7: Post Operative Complications

| Complication | Number | Percentage Affected |
| --- | --- | --- |
| Aspiration pneumonia | 3 | 2.3 |
| Bleeding – (no blood product administration) | 3 | 2.3 |
| Bleeding (blood products administered) | 6 | 4.7 |
| Cardiac arrythmia | 8 | 6.3 |
| Cardiac arrest | 3 | 2.3 |
| Hypotension | 6 | 4.7 |
| Hypertension | 2 | 1.6 |
| Regurgitation | 6 | 4.7 |
| Vomiting | 3 | 2.3 |
| Diarrhea | 3 | 2.3 |
| Acute Kidney Injury | 3 | 2.3 |
| Tachypnea | 3 | 2.3 |
| Hematoma – Surgical site | 2 | 1.7 |
| Inappetence | 5 | 1.6 |
| Hypoalbuminemia | 1 | 0.8 |
| Septic peritonitis | 1 | 0.8 |
| Peritoneal effusion | 1 | 0.8 |
| Bladder blood clot | 1 | 0.8 |
| Hypoglycemia | 1 | 0.8 |
| DIC | 1 | 0.8 |
| Gastrointestinal Ileus | 1 | 0.8 |
| Pancreatitis | 1 | 0.8 |

*Percentages are out of the total number of cases undergoing adrenalectomy for treatment of PCC (n = 128)*

## Outcomes of Group 3

Of the 52 cases that did not receive treatment with AA or adrenalectomy, 38.5% (n = 20) were recorded as alive at the time of data entry and 64% (n = 32) were dead. The median time from initial presentation to a referral center to the last recorded date of contact for cases that were alive was 27 (range, 0 – 545) days. A single case did not have a follow up date recorded. The reason for no treatment being administered was not recorded in 75% (n = 15) of these cases. Otherwise, reasons included one owner wishing to pursue palliative treatment only, three owners declining treatment outright and one case where AA treatment was recommended but had not been started at the time of follow up. Several cases recorded as alive (25%, n = 5) were lost to follow up following a discussion around treatment options and therefore ultimate outcomes or treatment approaches were unknown, this included one case where surgery was recommended, two cases where euthanasia was recommended or elected by the owner and two where AA treatment was recommended.

Of the 32 cases that were dead, the median time from initial presentation to a referral center to the last recorded date of contact was 7 (range, 0 – 1261) days. Half of these cases were euthanased (n = 16) due to clinical signs assumed to be related to their PCC. Euthanasia for other reasons was recorded as the cause of death in a further eight cases. Five cases experienced natural death, of which two cases were thought to be due to consequences of their PCC. In the remaining three cases, a reason for death was not recorded.

A survival curve of group 3 cases, using the date of presentation to a referral centre and the date of last contact is presented in supplementary figure S4, to indicate the degree of early censoring and death of cases in this group.


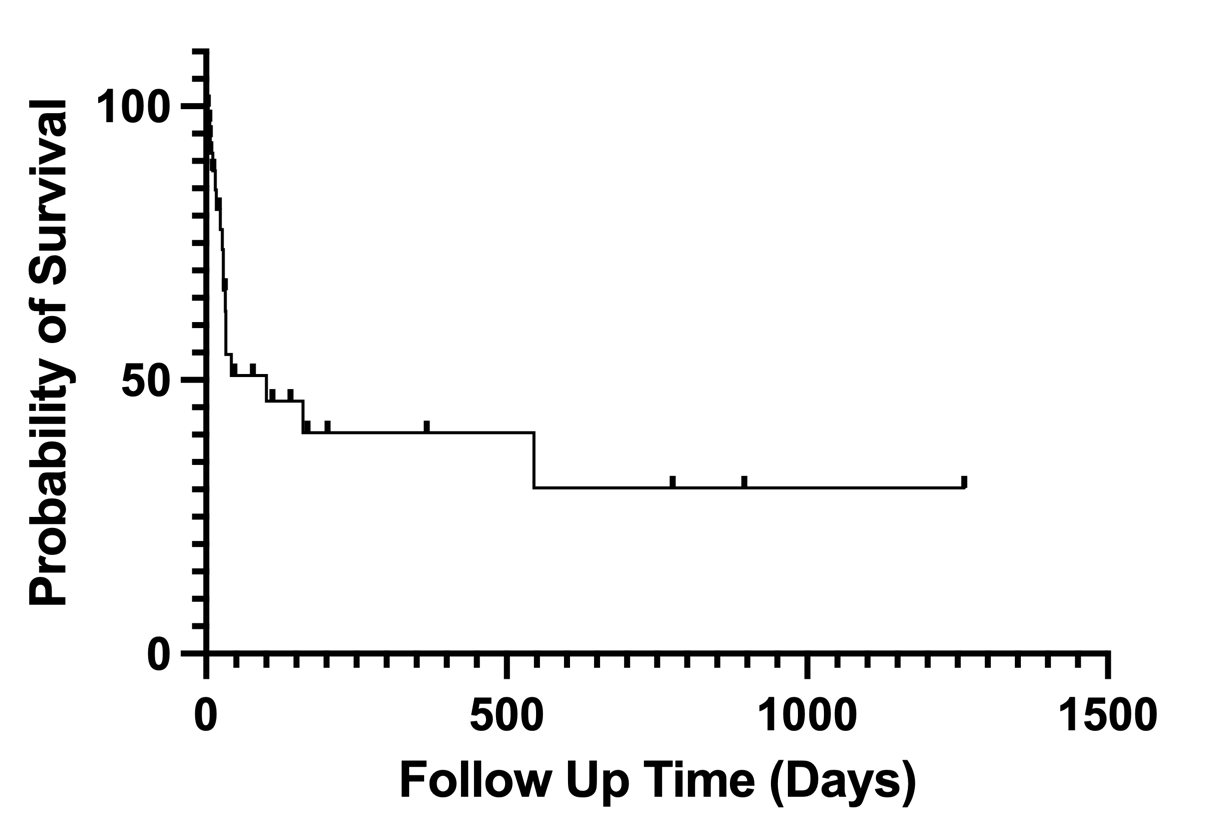


## Supplemental Figure S4: Kaplan-Meier estimate of follow up time for group 3 (no treatment) cases of canine PCC.

Vertical ticks represent censoring, vertical continuous lines represent events.

## Chemotherapy Details

In total 21 cases were recorded to have received some form of chemotherapy treatment. This included 15% (n = 11) cases within the AA treatment group (group 1), 7% (n = 9) cases within the surgical treatment group (group 2) and one case within group 3. Toceranib phosphate was the chemotherapy agent used in 9 cases in group 1, 4 cases in group 2 and 1 case within group 3. Other chemotherapy agents used in group 1 cases included one case that received masitinib and the remaining case did not have further details of chemotherapy recorded. In group 2, once case received doxorubicin, the remaining cases did not have details on chemotherapy agents recorded, likely due to the reasons for chemotherapy being related to other neoplasia (for example, one case with subcutaneous hemangiosarcoma, one case with splenic hemangiosarcoma, one case with mesothelioma and one with multicentric lymphoma).

## Supplemental Table S8: Causes of Death

|  | Group 1  n = 40 | Group 2  n = 28 |
| --- | --- | --- |
| Euthanasia  PCC  Not-PCC | 22 (55%)  10 (25%) | 9 (32%)  14 (50%) |
| Natural death  PCC  Not-PCC | 1 (3%)  7 (18%) | 27 (7%)  3 (11%) |

Supplemental Table S8: Distribution of causes of death for cases of canine PCC treated with AA alone (group 1) or treated surgically (group 2). PCC refers to death that was associated with consequences of PCC and not-PCC refers to death that was not considered to be associated with consequences of PCC.
